# Mucormycosis as the Elusive Cause of an Aortic Thrombus and Tissue-Obliterating Abscess

**DOI:** 10.1155/2019/4842150

**Published:** 2019-02-19

**Authors:** Shira R. Paul, Preston S. Gable

**Affiliations:** ^1^Department of Internal Medicine, Naval Hospital Camp Pendleton, Box 555191, Camp Pendleton, CA 92055, USA; ^2^Department of Hematology-Oncology, Naval Medical Center San Diego, 34800 Bob Wilson Drive, San Diego, CA 92134, USA

## Abstract

Invasive mucormycosis is an increasingly common cause of morbidity and mortality in hematologic malignancy patients. Early consideration of the diagnosis is essential in at-risk patients, exhibiting suggestive signs and symptoms. A 56-year-old female with acute myeloid leukemia initially presented with neutropenic fever before subsequently developing dense hemiplegia due to septic emboli to the spine and multifocal abscesses. These findings were later determined to be a result of a disseminated mucor infection and represented a rare manifestation of the disease. Despite the disseminated nature of the infection, identification of the causative organism was initially impeded by limitations in obtaining a tissue sample in a severely thrombocytopenic patient, as is common among hematologic malignancy patients. As a result of this limitation, diagnosis was ultimately made via PCR on bronchiolar lavage fluid. Early consideration of the diagnosis with prompt initiation of treatment is of utmost importance in this invasive infection. Further research is needed to identify and validate rapid, minimally invasive strategies for early diagnosis of mucormycosis.

## 1. Introduction

Mucormycosis is an uncommon fungal infection but occurs with greater frequency in hematologic malignancy patients. Presentation is varied and includes invasive pulmonary, sino-orbital, or disseminated disease. Diagnosis is challenging as blood cultures are typically negative, requiring histopathologic identification of the organism. We present the case of an acute myeloid leukemia patient with disseminated mucormycosis complicated by a large abscess in the left upper lobe obliterating the lung, chest wall, and spine, as well as an exceedingly rare aortic thrombus, which led to multifocal emboli.

## 2. Case

A 56-year-old female with acute myeloid leukemia (AML) was admitted for neutropenic fever after presenting with one day of fatigue. She denied other focal infectious symptoms. Four days prior, she was evaluated in the emergency department for left upper back pain, with work-up remarkable for neutropenia without fever and pulmonary artery CT showing a new focus of ground-glass attenuation within the superior segment of the left lower lobe. The back pain was presumed to be musculoskeletal due to reproducibility by palpation, and she was discharged with close follow-up. Past medical history was remarkable for treatment-related AML (due to prior breast cancer chemotherapy) diagnosed four months prior to this presentation. The treatment course was complicated by relapse one week after initial induction and prolonged neutropenia with neutropenic fever following both induction and reinduction. At time of presentation, medications included levofloxacin, sulfamethoxazole/trimethoprim, acyclovir, and voriconazole for prophylaxis.

On admission, she appeared mildly uncomfortable with tachycardia (heart rate 128), fever of 38.8°C, and otherwise normal exam. Lab evaluation showed pancytopenia with a WBC of 100 (no detectable neutrophils), hemoglobin of 8.5 g/dL, platelets of 22,000/mL^3^. Initial blood and urine cultures were negative. Chest CT showed worsening of previously identified left lower lobe ground-glass opacities, new consolidation of the left upper lobe, and fullness within the left paraspinal region. Vancomycin and meropenem were started for empiric treatment of neutropenic fever; voriconazole and acyclovir were continued. Early the morning after admission, the patient awoke with profound left leg weakness, with exam showing 0/5 strength in the left lower extremity, abnormal temperature sensation on the right side from T6 downwards, and urinary retention concerning for a thoracic spinal cord insult. Urgent MRI of the brain and spine showed patchy, nonenhancing regions of increased cord signal at T5 and T6-7, abnormal epidural enhancement from C6-7 level down to T7, and three distinct foci of acute cerebral ischemia, concerning for a multifocal embolic injury.

**Figure 1:**
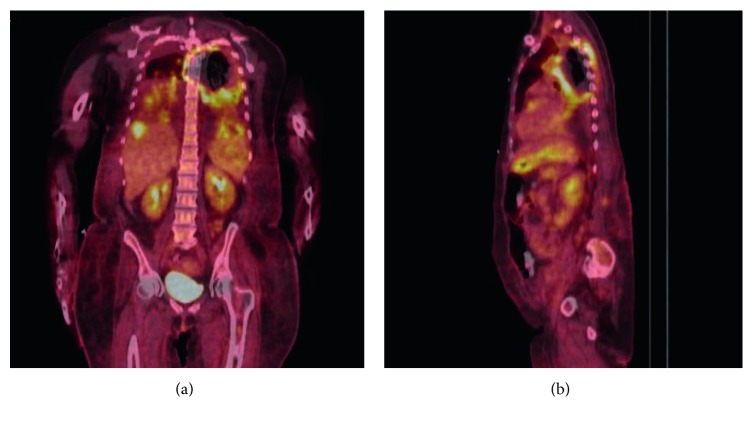
PET/CT images obtained in evaluation of aortic filling defect (coronal image (a); sagittal image (b)), showing hypometabolic activity within the spinal cord from T3 through T6 and a large (6.3 × 6.4 × 5.3 cm), ring-like area of FDG avidity with central, decreased avidity consistent with an abscess and central infarct of the left upper lobe, posterior chest wall, and adjacent thoracic spine vertebral bodies.

Over the following six days, she remained febrile and tachycardic despite further broadening of antibiotics to liposomal amphotericin and ganciclovir. Her neurologic deficits progressed to full paraplegia with bladder and bowel dysfunction. Extensive laboratory evaluation for a causative organism, including numerous blood cultures, remained negative. In an ongoing effort to identify an infectious etiology of the persistent neutropenic fever/pulmonary infection, CT chest/abdomen/pelvis, lumbar puncture, and bronchoscopy with bronchoalveolar lavage were performed. CT chest showed progression of consolidation, multiple, new, bilateral nodular opacities, and a filling defect within the descending aorta, concerning for development of an intraluminal thrombus. CT abdomen/pelvis also showed multiple, new hypodense lesions throughout the liver and spleen, concerning for developing abscesses. At this point, the aortic filling defect was felt to be the originating source of the likely septic embolic lesions identified throughout the brain, spine, chest, and abdomen. Due to the occurrence of the suspected septic embolic events while on broad-spectrum antibacterial and antifungal antibiotics, a PET/CT was pursued with a goal of better elucidating whether the aortic thrombus was infectious. The PET/CT showed hypometabolic activity within the spinal cord from T3 through T6, representing infarct, and a large (6.3 × 6.4 × 5.3 cm), ring-like area of FDG avidity involving the apical posterior left upper lobe and adjacent thoracic spine, with central, marked decreased avidity of included pulmonary parenchyma, thoracic vertebral bodies, spinal cord, and posterior chest wall (Figure 1).

Although definitive microbiologic diagnosis had not been made, the multifocal septic emboli and a large abscess were felt to be consistent with either an invasive fungal infection or Nocardiosis. After discussion of the PET/CT findings with the patient and her family, specifically the low likelihood of return of neurologic function and inability to do allogeneic hematopoietic cell transplant in a patient with such an infection, they chose to transition to home hospice care. Six days after discharge, results from broad-range fungal PCR performed on the bronchiolar lavage fluid sent to an outside institution detected *Rhizomucor pusillus* DNA.

## 3. Discussion

Mucormycosis is a rare, but increasingly prevalent cause of morbidity and mortality in patients with hematologic malignancy [1, 2]. Encompassing the *Rhizopus*, *Mucor*, *Rhizomucor*, and *Lichtheimia* species, Mucorales are ubiquitous, environmental fungi, and the third most common cause of invasive fungal infection complicating hematologic malignancy (after *Candida* and *Aspergillus*) [1]. Presentation is varied and may include pulmonary, sino-orbital, central nervous system, soft tissue, gastrointestinal, or disseminated disease and is associated with high risk of morality (prior estimates vary, as high as 40–70%) [3]. Risk factors for this opportunistic infection include hematologic malignancy, severe neutropenia, hematopoietic stem cell transplant, and uncontrolled diabetes [1, 3]. AML carries the greatest risk among hematologic malignancies, with incidence between 1 and 1.9% [1]. Early consideration of mucormycosis infection is critical as previous studies have shown significant increases in mortality in association with both delay in diagnosis [3] and delay in initiation of appropriate antibiotics [4]. Treatment requires an aggressive, multimodal approach with amphotericin-B, surgery, and control of underlying risk factors.

Despite importance of early recognition, diagnosis is often extraordinarily difficult as there is no available serologic assay (as in the case of galactomannan for *Aspergillus*), blood cultures are typically negative, and beta-D-glucan is not reliably positive [1]. Identification of a mucor infection requires histopathologic identification of the large diameter, irregularly branching hyphae, which is often complicated by difficulty obtaining tissue in severely thrombocytopenic hematologic malignancy patients.

This case highlights several challenges which are typical of the diagnosis of mucormycosis, as well as clinical features that are exceedingly rare, yet should prompt consideration of this diagnosis. In this case, mucor infection was considered early as a result of the patient's known risk factors (AML, prolonged neutropenia) and prophylaxis with voriconazole, to which mucorales are not susceptible. While the persistence of neutropenic fever was presumed to be related to the diffuse, septic embolic lesions identified on CT, invasive tissue biopsy of one of the lesions for microbiologic identification was not possible due to severe thrombocytopenia. Unique to this case was the identification of the aortic thrombus on CT—the likely source of the CNS, pulmonary, and abdominal embolic lesions. Angioinvasion is a key virulence factor in mucormycosis [5, 6]. While exceedingly rare, prior case reports have described formation of aortic thrombi leading to septic emboli to the spine [5] and to complete thrombotic occlusion of the aorta [6]. An additional case described massive hemorrhage from the aorta as a result of vascular invasion of the aorta from an adjacent pulmonary mucor infection [7]. We suspect that the large abscess, spanning multiple tissue planes (Figure 1) and the aortic thrombus were reflective of the angioinvasive nature of the disease. Unfortunately, all of the previously described cases of mucormycosis causing thrombosis or invasion of the aorta resulted in death of the patient, with microbiologic diagnosis only possible at time of autopsy.

Given the high mortality rate of mucormycosis and challenges of achieving histopathologic diagnosis, there is a critical need for a diagnostic approach which would allow for early diagnosis, less invasively. Previous groups have shown promise in distinguishing mucormycosis from other forms of invasive fungal infection (particularly aspergillosis) radiologically; however, this largely applies to pulmonary mucormycosis and lacks sensitivity [8]. Recently, several groups have also created assays for detection of mucormycosis via PCR. In a case series, Ino et al. performed PCR on peripheral blood of four hematologic malignancy patients, allowing identification of Mucorales DNA early in each patient's clinical course [9]. Scherer et al. similarly identified Mucorales DNA via PCR of bronchiolar lavage (BAL) fluid in 10 patients, additionally showing relative internal validation of results by identifying the same species' DNA via PCR of peripheral blood in 9 of the 10 patients [10]. While promising and briefly discussed as an emerging diagnostic modality in the 3rd European Conference on Infectious in Leukemia mucormycosis guidelines, PCR testing has yet to be standardized or validated for this purpose, as data are limited to small, retrospective studies and case reports. Additionally, the heightened sensitivity of the new assays in conjunction with limited validation could result in decrease in the specificity compared to the current gold standard, histopathology. In the retrospective study by Scherer et al., 2 of the 10 identified cases also had positive PCR for aspergillosis, creating question of whether there was true coinfection versus identification of a second mold, which was not the primary cause of the invasive fungal infection [10].

In this case, given the progressive clinical course despite broad-spectrum antibiotics and limitation in obtaining tissue, bronchoalveolar lavage fluid was evaluated with broad-spectrum PCR (primers directed to identify bacterial, fungal, and mycobacterial DNA), identifying *Rhizomucor pusillus* DNA. Ultimately, this patient's invasive mucormycosis progressed despite transition to liposomal amphotericin. Further validation and availability of PCR for diagnosis of mucormycosis could allow for earlier, more successful treatment of this highly fatal disease.

## 4. Conclusion

Mucormycosis is an increasingly prevalent cause of severe infection and mortality in hematologic malignancy patients. As a result of a proclivity for angioinvasion, mucor infection may cause an aortic thrombus and should be considered in the case of a severely immune-compromised hematologic malignancy patients presenting with multifocal embolic phenomenon. Although early diagnosis is critical, this is often complicated by difficulty in obtaining tissue in a thrombocytopenic patient. Hopefully, future studies will allow for validation of PCR or other rapidly and easily obtained diagnostic modality for this purpose.
